# Effects of slit dual-frequency ultrasound-assisted pulping on the structure, functional properties and antioxidant activity of *Lycium barbarum* proteins and in situ real-time monitoring process

**DOI:** 10.1016/j.ultsonch.2023.106696

**Published:** 2023-11-17

**Authors:** Shuhan Liu, Tianyu Kong, Yuqin Feng, Yanli Fan, Junwei Yu, Yuqing Duan, Meihong Cai, Kai Hu, Haile Ma, Haihui Zhang

**Affiliations:** a School of Food and Biological Engineering, Jiangsu University, Zhenjiang 212013, China; b School of Food & Wine, Ningxia University, Yinchuan 750021, China; c Ningxia Zhongning Goji Industry Innovation Research Institute, Zhongning 755100, China; d Institute of Food Physical Processing, Jiangsu University, Zhenjiang 212013, China

**Keywords:** *Lycium barbarum* pulp, Protein, Antioxidant, Functional properties, Dissolution kinetic model, In situ real-time monitoring

## Abstract

To improve the protein dissolution rate and the quality of fresh *Lycium barbarum* pulp (LBP), we optimized the slit dual-frequency ultrasound-assisted pulping process, explored the dissolution kinetics of *Lycium barbarum* protein (LBPr), and established a near-infrared spectroscopy in situ real-time monitoring model for LBPr dissolution through spectral information analysis and chemometric methods. The results showed that under optimal conditions (dual-frequency 28–33 kHz, 300 W, 31 min, 40 °C, interval ratio 5:2 s/s), ultrasonic treatment not only significantly increased LBPr dissolution rate (increased by 71.48 %, *p* < 0.05), improved other nutrient contents and color, but also reduced the protein particle size, changed the amino acid composition ratio and protein structure, and increased the surface hydrophobicity, zeta potential, and free sulfhydryl content of protein, as well as the antioxidant activity of LBPr. In addition, ultrasonication significantly improved the functional properties of the protein, including thermal stability, foaming, emulsification and oil absorption capacity. Furthermore, the real-time monitoring model of the dissolution process was able to quantitatively predict the dissolution rate of LBPr with good calibration and prediction performance (*Rc* = 0.9835, *RMSECV* = 2.174, *Rp* = 0.9841, *RMSEP* = 1.206). These findings indicated that dual-frequency ultrasound has great potential to improve the quality of LBP and may provide a theoretical basis for the establishment of an intelligent control system in the industrialized production of LBP and the functional development of LBPr.

## Highlights

• Slit dual-frequency ultrasound-assisted pulping of *Lycium barbarum* was optimized.
• Optimized processing improved *Lycium barbarum* pulp quality and protein dissolution.
• Ultrasound improved functional & antioxidant properties of *Lycium barbarum* protein.
• A model for dissolution kinetics of *Lycium barbarum* protein was developed.
• An in situ real-time monitoring model of *Lycium barbarum* pulping was established.

## Graphical abstract

**Figure:**
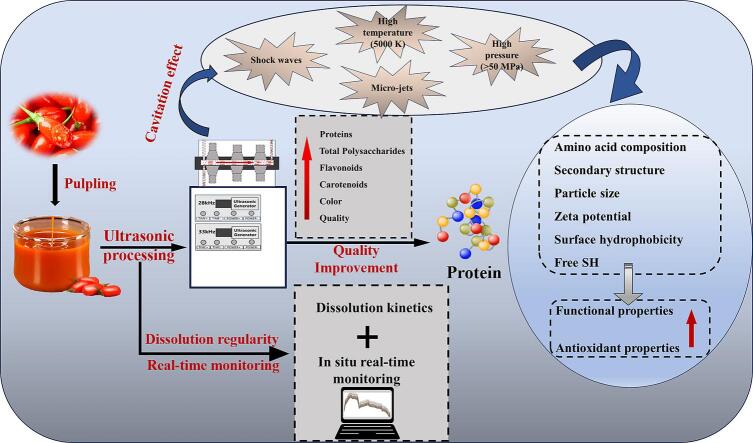

## 1 Introduction

*Lycium barbarum* is a perennial shrub of the genus *Lycium* in the family *Solanaceae*, which grows in temperate to subtropical regions of Eurasia, Australia, and North and South America [1]. In China, *Lycium barbarum* has been cultivated and used as a medicine and functional food for thousands of years [2]. It is favored by mass consumers due to its abundance of various bioactive secondary metabolites. At present, there are a wide variety of deep-processed *Lycium barbarum* products in the market, mainly including fresh berries, drinking juice, matching tea [3], [4] or adding to the assorted nuts, cereals, muffins, etc*.* [5], [6], [7]. *Lycium barbarum* pulp (LBP) is directly processed from the fresh fruits, which has become a popular product among consumers due to its unique taste and rich nutritional value. It is not only rich in polysaccharides, flavonoids, polyphenols, carotenoids substances and other active ingredients, but also contains proteins, peptides [8]. Studies have shown that *Lycium barbarum* protein (LBPr) possesses antihypertensive, antitumor, and immunomodulatory effects, making it a promising source of biologically active proteins with research potential [9], [10].

Low dissolution rate of nutrients during mechanical juicing and high degradation of heat-sensitive bioactive compounds are the two challenges in traditional LBP pulping process [11]. Therefore, there is an urgent need for a safe and efficient way to enhance the quality of LBP. Ultrasound is considered a non-thermal physical processing technology, which has received great attention in recent years due to its ability to maintain the freshness, flavor, and nutrients of raw material with low energy consumption [12], [13]. Existing data show that ultrasonic treatment is beneficial for increasing the bioactive compounds content (polysaccharides, proteins, polyphenols) and antioxidant activity in kiwifruit juice [14], blackberry juice [15], and strawberry juice [16], thereby improving product quality. Ultrasonic cavitation generates shear flows, microjets, turbulence, and excitation, that provides driving force to break cell wall, resulting in solvent penetration and rapid dissolution of protein and other substances [17]. However, conventional ultrasound processing is usually performed by probe-type or bath-type ultrasound device with fixed single frequency, which has limitations such as small scale, and uneven acoustic field [18]. Notably, the narrow cavity of slit ultrasonic equipment is easy to produce a large area of high sound pressure, and the flow field speed is fast, which is more conducive to reaching the cavitation threshold [29]. In addition, ultrasound frequency modes have significant impacts on the cavitation intensity [19], compared with single frequency ultrasound, multi-frequency ultrasound has the advantages of wave superposition, uniform dispersion, promoting mass transfer, energy concentration [20], [21]. Studies have shown that slit dual-frequency ultrasound could effectively overcome cavitation shielding, therefore a large amount of energy will be released immediately when the cavitation bubbles burst [22], and significantly improve the yield of *Lycium barbarum* polysaccharide[29], *Chlorella pyrenoidosa* protein[100] and watermelon seed protein[71], and enhance their functional properties and antioxidant activities, outperforming single/triple frequency ultrasound treatment. In the process, it may be accompanied by the changes in secondary structure of protein molecule and the reduction of particle size, which are the important factors to improve the functional properties and biological activity of protein [23], [24]. Based on these, we speculated that slit dual-frequency ultrasound may promote the dissolution of LBPr as well as the other nutrients, change the molecular structure of LBPr, and improve some properties, thereby enhancing the nutritional value of LBP.

Conventional monitoring methods based on instrumental analysis are complicated and time-consuming, making it impossible to realize real-time dynamic monitoring of protein dissolution [25]. Therefore, it is particularly important to explore a fast, efficient and continuous monitoring method for LBPr dissolution process. Kinetic model can reflect the basic trend to a certain extent, which helps to understand the intrinsic mechanism, effectively predicts and optimizes the enhanced dissolution process. In addition, near-infrared (NIR) spectroscopy could realize on-line monitoring by detecting the characteristic information of C-H, O-H, N-H and other hydrogen-containing groups in the molecules of organic substances within the NIR spectral region of the octave and combined-frequency telescopic vibrations [26]. Due to its advantages of low cost, high efficiency, green environmental protection, and on-line non-destructive testing, NIR has developed into an emerging means of analysis and research in recent years. Currently, NIR spectroscopy has been widely used in food industry, such as monitoring the extraction process of proteins and polysaccharides [20], [27], the preparation of active peptides and chemical drugs [21], [28], and the extraction of *Lycium barbarum* polysaccharides [29]. With the development of miniature NIR fibre-optic probes and their spectroscopic systems, NIR technology can be conveniently applied to the ultrasonic fortification process of LBP, demonstrating great potential in the intelligent production of high-quality and highly active LBP.

Therefore, in order to improve the dissolution of LBPr and other nutrients, as well as the color of LBP, the slit dual-frequency ultrasound-assisted pulping of fresh *Lycium barbarum* was optimized and a kinetic model for LBPr dissolution was established. In addition, the effects of ultrasonic treatment on the molecular structure, functional properties, microstructure, and antioxidant activity of LBPr were investigated. Moreover, an in situ real-time monitoring model of NIF spectra was established by collecting real-time spectral information of LBP throughout the pulping process. These studies may provide a theoretical basis for intelligent control of LBPr dissolution during slit dual-frequency-assisted fresh fruit pulping.

## 2 Materials and methods

### 2.1 Materials and chemicals

Fresh fruits of *L. barbarum* (provided by Zhongning, Ningxia, China). Sodium hydroxide (NaOH), Folin-Ciocalteu reagent, 2,2′-azinobis (3-ethylbenzothiazoline-6-sulphonic acid) (ABTS^+^•), 1,1-Diphenyl-2-picrylhydrazyl radical 2,2-Diphenyl-1-(2,4,6-trinitrophenyl) hydrazyl.

(DPPH•) were purchased from Macklin (Shanghai, China). 8-Anilino-1-naphthalenesulfonic acid (ANS) and 5, 5′-dithio-bis-(2-nitrobenzoic acid) (DNTB) were purchased from Sigma-Aldrich (Shanghai, China). Other chemicals and solvents used in the experiments were of analytical grade.

### 2.2 Process optimization of slit dual-frequency ultrasound fortification of LBP

#### 2.2.1 Single factor experiment

**Fig. 1:**
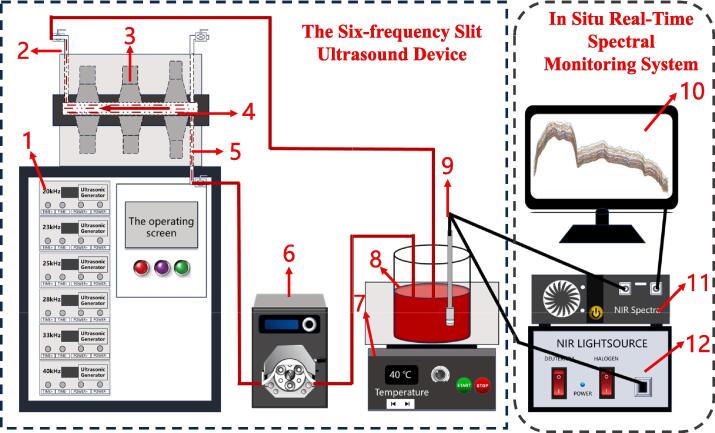
Ultrasound equipment and in situ real-time monitoring system. (1) ultrasonic generators; (2) exit port; (3) ultrasonic transducer; (4) slit cavity; (5) injection port; (6) peristaltic pump; (7) magnetic stirring water bath; (8) LBP; (9) fiber optic probe; (10) data analysis platform; (11) near-infrared spectroscopy; (12) light source.

Fresh *Lycium barbarum* fruits were washed with distilled water and ultrasonicated with a slit ultrasonic reaction generator (developed by Jiangsu University). The device is shown in Fig. 1, which has the advantages of uniform acoustic field distribution, low energy consumption, and energy concentration [30]. The ultrasonic working mode in this study was simultaneous dual-frequency (28–33 kHz, two frequencies of ultrasound working at the same time) mode, and the protein dissolution rate in LBP was used as an index to conduct a one-factor test of the enhanced pulping process to preliminarily identify the ultrasonic power (100, 200, 300, 400, 500 W), ultrasonic treatment temperature (20, 30, 40, 50, 60 °C), time (10, 20, 30, 40, 50 min), and intermittent ratio (the ratio of ultrasonic working time to interstitial time, 5:1, 5:2, 5:3, 5:4, 5:5 s/s) on the protein dissolution rate of LBP.

#### 2.2.2 Response surface methodology (RSM) experiments

According to the single-factor experiment results, RSM was further used to optimize important variables (ultrasound power, time, and temperature). As shown in Table 1, the whole design consisted of 17 experiments, each experimental set was repeated three times and averaged to facilitate subsequent data analysis. The regression fitting model was represented by the following second-order polynomial:

(1) $$Y=A_{0}+\sum_{i=1}^{3}A_{i}X_{i}+\sum_{i=1}^{3}A_{\mathrm{ii}}X_{i}^{2}+\sum_{i=1}^{2}\sum_{j>1}^{3}A_{\mathrm{ij}}X_{i}X_{j}$$

where *Y* is the predicted response value; *A_i_*, *A_ii_*, and *A_ij_* are the primary term coefficients, quadratic term coefficients, and interaction term coefficients, respectively; and *X_i_* and *X_j_* are independent variables.

**Table 1:** RSM Box-Behnken design and results for protein dissolution rate (*Y*).

Run	A (Time)	B (Temperature)	C (Power)	*Y* (%)
1	20	30	300	11.49
2	40	30	300	11.67
3	20	50	300	11.31
4	40	50	300	11.76
5	20	40	200	9.01
6	40	40	200	9.89
7	20	40	400	10.74
8	40	40	400	10.51
9	30	30	200	9.96
10	30	50	200	9.76
11	30	30	400	11.27
12	30	50	400	10.82
13	30	40	300	13.35
14	30	40	300	13.29
15	30	40	300	13.32
16	30	40	300	13.33
17	30	40	300	13.19

### 2.3 Preparation of LBPr

The LBP was sonicated and immediately centrifuged (10000 rpm, 15 min), filtered, and then collected the supernatant. Protein content was determined by the Bradford method using bovine serum protein as standard [31]. The supernatant was adjusted to pH 3.5 with 1 mol/L HCl, allowed to settle overnight, centrifuged (4000 rpm, 25 min) and the sediment was collected. The precipitate was redissolved with distilled water and the pH was adjusted to 7.0. The distilled water was changed every 6 h for a total of 72 h of dialysis, and the LBPr (ultrasonic treatment: U-LBPr; traditional processing: T-LBPr) was obtained by lyophilization.

### 2.4 Determination of nutrients in LBP

The total sugar content was determined by the phenol sulfate method [32], using D-glucose as the standard. Reducing sugars were measured by the DNS colorimetric method [33], and the polysaccharide content was determined by subtracting the reducing sugar content. Polyphenolic and flavonoid contents of LBP were determined by Folin-Ciocalteu method and aluminum chloride colorimetric method [34], [35]. Carotenoids were determined by the method of Akter M.K [36]. The contents of different nutrients in LBP before and after ultrasonic treatment were determined in detail and further compared and analyzed.

### 2.5 Determination of LBP color

In this study, the color changes of the samples were evaluated by a portable colorimeter (CR-300 Chroma, Minolta, Japan). CIELab parameters including L*, a*, and b* of each sample were recorded. The total color difference (*ΔE*), chroma (*C*), and hue angle (*h*) were obtained from Eqs. (2)–(4), respectively [37].

(2) $$\Delta E=\sqrt{{\left(L*-L_{0}*\right)}^{2}+{\left(a*-a_{0}*\right)}^{2}+{\left(b*-b_{0}*\right)}^{2}}$$

(3) $$C=\sqrt{{a*}^{2}+{b*}^{2}}$$

(4) $$h=\tan^{-1}\left(b*/a*\right)$$

### 2.6 Dissolution kinetics of LBPr

In order to gain further insight into the mass transfer mechanism of LBPr, a nonstationary diffusion model based on Fick's second law was developed to investigate the effects of different ultrasonic frequency modes (28–33, 23–40, 25–40, 25–28, 28–40 kHz), power (100, 150, 200, 250, and 300 W), and time (5, 10, 15, 20, 25, 30, 35, 40, 45, and 50 min) on the dissolution rate of LBPr in raw pulp. The model was applied to describe the kinetic process of LBPr dissolution.

Some assumptions must be made before modeling can proceed [38]. (a) the *Lycium barbarum* particles are ideal spherical particles and maintain their shape unchanged, with a uniform distribution of internal proteins; (b) the extraction suspension is well mixed due to microturbulence and cavitation bubbles, so that the external resistance to mass transfer is negligible [39]. Based on above hypothesis, the Fourier transform solution yields the kinetic model equation of Fick's second law, which could be expressed as [40]:

(5) $$\frac{\left(C_{e}-C_{t}\right)}{\left(C_{e}{-C}_{0}\right)}=\left(6/\pi^{2}\right)\sum_{n=1}^{\infty }\left\{\left(1/n^{2}\right)exp\left[-{\left(n\pi /R\right)}^{2}D_{s}t\right]\right\}$$

where *C_t_* is the mass concentration of protein in the main body of the solution at any time *t*, mg/mL; *C_0_* and *C_e_* are the mass concentrations of protein in the solution at the initial time *t* and when the dissolution reaches equilibrium, mg/mL; *D_S_* is the effective diffusion coefficient, which indicates the diffusion ability of the substance in the medium, mm^2^/s.

Since the higher concentration term is negligible, the above equation holds for *n* = 1[41].

(6) $$\left(C_{e}-C\right)/\left(C_{e}{-C}_{0}\right)=\left(6/\pi^{2}\right)\sum_{n=1}^{\infty }\left\{exp\left[-\pi^{2}D_{s}t/R^{2}\right]\right\}$$

When *C_0_* = 0, Eq. (6) could be obtained.

(7) $$ln\left[C_{e}/\left(C_{e}-C_{t}\right)\right]=kt+b$$

(8) $$k=\pi^{2}D_{s}/R^{2}$$

where *k* denotes the extraction rate constant, which reflects how fast the substance diffuses through the medium, s^−1^; and *b* denotes the intercept. The relative raffinate rate (*y*) and half-life period ($t_{1/2}$) are described as follows:

$$Relative\;raffinate\;rate:\;y=\left(C_{e}-C_{t}\right)/C_{e}\;=\;\left(6/\pi^{2}\right)exp\left(-kt\right)$$

$$Half-life\;period:\;t_{1/2}=\left[ln2-ln\left(\frac{\pi^{2}}{6}\right)\right]/k$$

### 2.7 Structural characteristics

#### 2.7.1 Sodium dodecyl sulfate–polyacrylamide gel electrophoresis (SDS-PAGE)

SDS-PAGE of T-LBPr and U-LBPr was performed according to the protocol of Ruan et al [42].

#### 2.7.2 Total amino acids (AA)

Amino acid profiles were determined using an automated amino acid analyzer (S-433D, Sykam Co., Munich, Germany) according to the method of Jiang et al. with some modifications [43]. The sample (25 mg) was placed in a vacuum hydrolysis tube, and 10 mL of 6 mol/L HCl solution was added for acidolysis, which was sealed by filling with nitrogen, and the reaction was carried out at 110 °C ± 1 °C for 24 h. At the end of the reaction, the sample was filtered and diluted to 25 mL with ultrapure water. 1 mL of the filtrate was collected, dried at 50 °C, and solubilized on the apparatus. Amino acid content was calculated as g amino acid/100 g protein.

#### 2.7.3 Uv–visible (UV–Vis) spectroscopy

LBPr was dissolved in distilled water to prepare a 0.1 mg/mL solution, and the UV–Vis spectroscopy was obtained by scanning the solution between 200 nm and 400 nm at room temperature using a Varian Cary 100 UV–Vis Spectrophotometer (Varian Inc., CA, USA), while distilled water was used as a blank control.

#### 2.7.4 Fluorescence spectroscopy

Endogenous fluorescence analysis was performed using a fluorescence spectrophotometer (Model Cary Eclipse, Varian Inc., CA, USA) according to Xiong [44]. LBPr solution at a concentration of 0.1 mg/mL, with excitation at 280 nm, emission at 300–500 nm, and slit width of 5 nm, was scanned with distilled water as a control.

#### 2.7.5 Fourier transform infrared spectroscopy (FTIR)

A Fourier transform infrared spectrometer (Nicolet iS50, Thermo Fischer Scientific, MA, USA) was used for the acquisition of spectra. The dried sample was mixed with specific KBr, ground, pressed, and placed on a sample holder for spectral scanning in the range of 500 to 4000 cm^−1^ with a spectral resolution of 4 cm^−1^ and 32 cumulative scans.

The peaks in the amide I region (1700–1600 cm^−1^) band were fitted using the Fourier autoregressive (FSD) and second-order derivative analysis methods, and the integral of each peak was divided by sum of all identified peaks to determine percentage of each structure [45].

#### 2.7.6 Particle size and zeta potential

The particle size and zeta potential of LBPr were determined at 25 °C with a laser particle sizer (Nano ZS, Malvern Instrument Ltd., UK) as described by Wang Y [46]. LBPr samples (0.1 mg/mL) were prepared with deionized water for determination.

#### 2.7.7 Surface hydrophobicity (S_0_)

S_0_ refers to the initial slope of fluorescence intensity versus protein concentration (calculated by linear regression analysis), which was measured by the method of Jarpa-Parra using ANS as a fluorescent probe [47].

#### 2.7.8 Free SH contents

The amount of free SH in LBPr was determined according to a previous method described in literature [48].

#### 2.7.9 Microstructure

The microstructure of LBPr was further investigated by scanning electron microscopy according to the method of Flores-Jiménez N T [49].

### 2.8 Functional properties

#### 2.8.1 Differential scanning calorimetry (DSC)

Thermal properties of T-LBPr and U-LBPr were determined by differential scanning calorimeter (DSC 214 Polyma, Netzsch, Germany). According to the method of Das M [50], 5 mg sample was enclosed in a sealed aluminum pot and operated with air (empty pot) as a reference, with scanning temperatures ranging from 25 °C to 100 °C and a heating rate of 10 °C/min. Enthalpy changes (*ΔH*), peak denaturation temperatures (*Tp*), and DSC curves were obtained for U-LBPr and T-LBPr.

#### 2.8.2 Protein content and solubility

Crude protein content in raw material (7.26 g/100 g), T-LBPr and U-LBPr were determined by Kjeldahl method (Velp UDK159, Italy, recovery ≥ 99.5 %). Accurately weighed 50 mg of protein sample was dissolved in 25 mL of distilled water, stirred magnetically for 30 min, and the pH value of the solution was adjusted to 2.0–7.0 with 0.1 mol/L HCl or 0.1 mol/L NaOH solution, stirred at room temperature for 20 min, then centrifuged at 8,000 rpm for 15 min, and the protein content of the supernatant was measured by the Bradford method [51]. The calculation formula was as follows:

(11) $$Proteinsolubility\left(\%\right)=\frac{\mathrm{Proteininthesupernatant}}{\mathrm{Totalprotein}}\times 100$$

#### 2.8.3 Emulsion activity and stability indices

The emulsion activity and stability index of LBPr were determined as a reported method with slight modification [52]. Briefly, 1 g of protein sample was thoroughly mixed with 100 mL of distilled water, the pH was adjusted to 2.0–7.0 with 1.0 mol/L HCl and 1.0 mol/L NaOH, immediately removed 50 µL of the solution from the bottom of the tube, added 5 mL of corn oil, mixed at high speed for 2 min at 15000 rpm, then transfered to another tube containing 5 mL of 0.1 % SDS solution and mix well. After mixing, 50 μL of the solution was immediately aspirated from the bottom of the tube and transferred to a centrifuge tube containing 5 mL of 0.1 % SDS solution and mixed well. The absorbance *A_0_* was measured at 500 nm. After 30 min, the absorbance *A_30_* was measured and calculated with the following formula:

(12) $$EAI\left(m^{2}/g\right)=\frac{2\times 2.303\times D\times A_{0}}{C\times \Phi \times 10^{4}}$$

(13) $$ESI\left(min\right)=\frac{A_{0}}{A_{0}-A_{30}}\times 10$$

where *D* is the dilution factor; *A_0_* and *A_30_* are the absorbance at 0 and 30 min; respectively, *C* is the protein concentration, g/mL; and *Φ* is the oil volume fraction of the emulsion

#### 2.8.4 Foaming capacity and stability

The method of Aydemir was used and slightly modified [53]. In brief, the pH of sample solution with certain concentration, was adjusted to 2.0–7.0 with 1.0 mol/L HCl and 1.0 mol/L NaOH, then stirred at 15000 rpm for 2 min and measured foam volume *V_0_* at stirring stop; after 30 min, the foam volume *V_30_* was determined. The calculation formula is as follows:

(14) $$FC\left(\%\right)=\frac{V_{0}}{V}\times 100$$

(15) $$FS\left(\%\right)=\frac{V_{0}}{V_{30}}\times 100$$

where *V_0_* is the foaming volume at the mixing cut-off moment, mL; *V_30_* is the foaming volume after 30 min, mL; *V* is the initial volume, mL.

#### 2.8.5 Water absorption capacity and oil absorption capacity

The water absorption capacity (WAC) and oil absorption capacity (OAC) were determined as a reported method [54]. The formula is as follows, and the results are expressed in grams of water or oil absorbed per gram of protein.

(16) $$\left(WAC/OAC\right)=\frac{W_{2}-W_{1}}{W_{0}}$$

where *W_0_* is the amount of protein, g; *W_1_* is the centrifuge tube and the amount of protein, g; *W_2_* is the centrifuge tube and the amount of protein after removing the supernatant, g.

### 2.9 Antioxidant activity assay

#### 2.9.1 DPPH• radical scavenging activity

The DPPH• radical scavenging activity was performed according to the method of Hu [55]. The samples (1 mL; 0.1, 0.8, 1.6, 2.4, 3.2 mg/mL) were incubated with 250 μL of DPPH• solution for 30 min at room temperature in the dark, and the absorbance was measured at 517 nm using a microplate reader (Infinite M200Pro, Tecan, Austria).

#### 2.9.2 ABTS+• radical scavenging activity

The ABTS+• scavenging activity was determined according to the method of Zhu [56]. Different concentrations of LBPr (0.1, 0.8, 1.6, 2.4, and 3.2 mg/mL) were mixed with ABTS+• solution (1:1, v/v) and reacted for 10 min in the dark at room temperature, and the absorbance was measured at 734 nm.

#### 2.9.3 Reducing power

The reducing power of LBPr was determined according to a previous method with minor modification [57]. LBPr, with different concentrations (0.1, 0.8, 1.6, 2.4, 3.2 mg/mL), reacted with the reagent and the absorbance was measured at 700 nm.

### 2.10 NIR spectroscopy for real-time monitoring of LBPr dissolution

The real-time NIR in-situ monitoring system for ultrasonic assisted LBP preparation is shown in Fig. 1. The system mainly consists of a six-frequency slit ultrasound device, a miniature portable Ocean Optics NIR spectrometer (NIRQUEST256-2. 5, Ocean Optics, Largo, FL, USA), a tungsten halogen lamp light source (DH-2000-BAL, Ocean Optics, American), a liquid-immersible tranreflective fiber optic probe (TP300 immersion optical fiber probe, Ocean Optics, American), and a signal acquisition system.

Under the optimal conditions of ultrasound-assisted pulp preparation, the samples were collected every 30 s, and the average of three consecutive scans was taken as the raw spectra of the samples. The spectral range was 900 ∼ 2500 nm, total 256 spectra were acquired, the resolution was 6.4 nm, the optical range was 4 mm, and distilled water was used as the background under the same experimental conditions. A total of 100 samples were collected throughout the sonication process, and the supernatant was then collected by centrifugation for 15 min (10000 rpm) and stored at −4 °C for protein content determination.

### 2.11 Statistical analysis

All experiments were carried out in triplicate, and the data were expressed as the mean ± standard deviation. SPSS 26.0 software was used for one-way ANOVA, and *p* < 0.05 was consider to be statistically significant. The figures were plotted with Origin 2018 software, the near infrared spectrogram processing and model establishment were analyzed with Matlab R2020a software.

## 3 Results and discussion

### 3.1 Single factor experiment

**Fig. 2:**
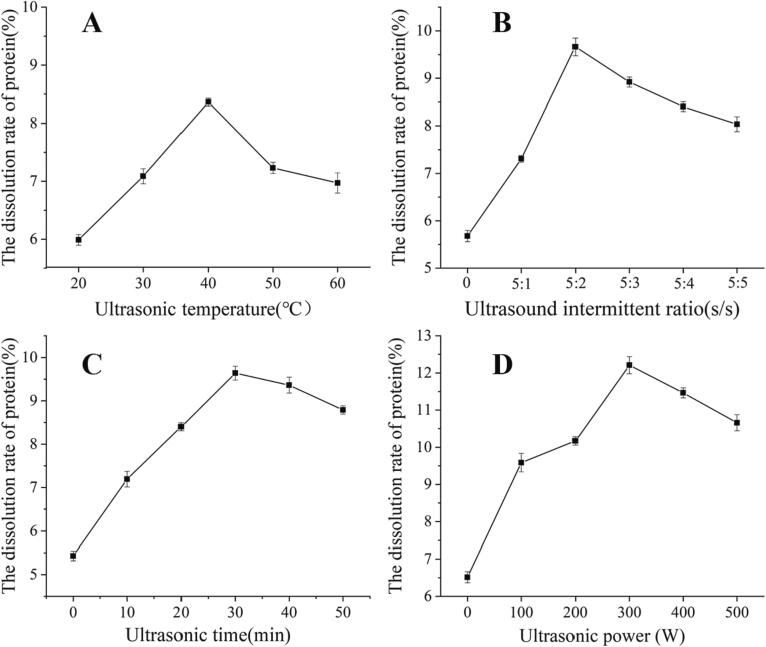
Effect of different treatment factors on the dissolution rate of *Lycium barbarum* protein. Ultrasonic temperature (A), Ultrasound intermittent ratio (B), Ultrasonic time (C), Ultrasonic power (D).

The dissolution of LBPr varies greatly due to different sonication parameters. As shown in Fig. 2A, as the thermal movement of protein molecules intensifies, the dissolution rate of LBPr increases with the increase of temperature [58], however, when the temperature exceeds 40 °C, the proteins may be denatured and form less water-soluble agglomerates, resulting in a decrease in the dissolution rate [59]. In addition, intermittent ultrasound is more efficient than continuous ultrasound [60]. At a sonication interval ratio of 5:2 (s/s), the dissolution rate reached a maximum value of 9.66 %. This could be explained by the fact that more cavitation bubbles are generated at this sonication interval ratio, which enhances the destruction of the cell wall and thus releases proteins [61]. Similarly, as the ultrasound time and power increases, the dissolution rate shows a tendency to increase and then decrease, indicating that the appropriate ultrasound time and power could help to enhance the cavitation effect and accelerate the dissolution of proteins [62]. However, the excessive cavitation effect causes the formation of disulfide bonds between cysteine residues, and the generated hydroxyl radicals stimulated protein aggregation, resulting in the decrease of protein dissolution rate [63]. Based on these, the optimal level of each factor was preliminarily determined and then further optimized by response surface methodology.

### 3.2 Fitting of response surface mathematical models

To achieve the maximum protein dissolution in LBP, the ultrasonic power, ultrasonic time, and ultrasonic temperature were optimized through Box-Behnken design. As shown in Table 1, based on existing data, a regression analysis was performed to establish a second-order polynomial equation containing linear, interaction, and quadratic terms. The relationship between the process variables and the response value (LBPr dissolution rate) is shown below:

$$Y=13.30+0.16A-0.092B+0.59C+0.067AB-0.28AC-0.062BC-1.08A^{2}-0.66B^{2}-2.18C^{2}$$

where *A*, *B* and *C* are the ultrasound time, ultrasound temperature and ultrasound power, respectively, and *Y* is the LBPr dissolution rate. Analysis of variance (ANOVA) is used to evaluate the experimental results and the statistical confidence of the developed model, and the results are shown in Table 2. The value of F was 457.00, *p* < 0.05, and the goodness of fit (*R^2^* = 0.9961 and *R^2^_adj_* = 0.9799) suggests that the model is highly significant and can accurately describe the relationship between the variables and the responses [64]. As the value of each factor increases, the protein dissolution rate tends to increase and then decrease, and there is a maximum point on the response surface, with ultrasonic power being particularly significant. Moreover, a significant interaction exists between ultrasound time and ultrasound power (*p* < 0.05).

**Table 2:** ANOVA of the response surface quadratic model of *Lycium barbarum* protein dissolution rate.

Source	Sum of Squares	df	Mean Square	F-Value	*P*-value	Significance
Model	32.45	9	3.61	457.00	< 0.0001	**
A	0.20	1	0.20	25.96	0.0014	**
B	0.068	1	0.068	8.68	0.0215	*
C	2.78	1	2.78	353.02	0.0001	**
AB	0.018	1	0.018	2.31	0.1723	ns
AC	0.31	1	0.31	39.05	0.0004	**
BC	0.016	1	0.016	1.98	0.2021	ns
A^2^	4.88	1	4.88	618.82	< 0.0001	**
B^2^	1.84	1	1.84	233.74	< 0.0001	**
C^2^	20.04	1	20.04	2540.67	< 0.0001	**
Residual	0.055	7				
Lack of Fit	0.039	3	0.013	3.29	0.1399	ns
Pure Error	0.016	4				
Cor Total	32.50	16				
R^2^ = 0.9961	R^2^_adj_ = 0.9799	C.V. % = 0.78 %				

Note: * significant (*p* ＜ 0.05); ** very significant (*p* ＜ 0.01); ns: not significant (*p* ＞ 0.05）.

According to the developed mathematical model, the optimal conditions are: ultrasonic power 316.35 W, ultrasonic temperature 39.58 °C, ultrasonic time 30.74 min, ultrasonic interval ratio 5/2 s/s, ultrasonic frequency 28–33 kHz, and the predicted LBPr dissolution rate of 13.34 %. For ease of operation, the ultrasonic power, processing time, and temperature were respectively adjusted to 300 W, 40 °C, and 31 min, while other parameters remained unchanged. Under these conditions, the LBPr dissolution rate was 13.25 %, compared with traditional pulping method, the protein content of LBP increased by 71.48 % after the ultrasonic treatment.

### 3.3 Effect of ultrasonic treatment on nutrient composition and color of LBP

Nutrient content and color attributes are considered important criteria for evaluating the quality of fruit juice or related products to satisfy the requirements of consumers [35]. Under optimal ultrasound assisted pulping conditions, the changes in the nutritional content and color of LBP were determined. As shown in Table 3, after sonication, the total polysaccharides, polyphenols, flavonoids, and carotenoids in LBP increased by 52.85 %, 72.97 %, 43.49 % and 21.49 %, respectively. In addition, the values of a*, b* and Δ*E* significantly improved, but no significant differences in brightness (L *) values were found. Chroma (*C*) of the LBP increased significantly, which was caused by the increase of a* and b*. The reason for the decrease in hue (*h*) values may be the hydroxyl group formed after ultrasonic treatment, resulting in an increase in red intensity [11]. These results indicated that ultrasound helps to promote the dissolution of active ingredients while also improving the color of LBP.

**Table 3:** Effect of Ultrasound on nutritional composition and color of *Lycium barbarum* pulp.

	Control	US
Total Polysaccharides(mg/g)	465.12 ± 0.84^a^	710.95 ± 0.67^b^
Reducing-sugar(mg/g)	178.54 ± 1.82^a^	224.15 ± 0.94^b^
Flavonoid(mg/g)	2.37 ± 0.18^a^	3.61 ± 0.42^b^
Carotenoid(mg/g)	0.42 ± 0.03^a^	0.54 ± 0.07^b^
Protein(mg/g)	22.30 ± 0.21^a^	38.16 ± 0.17^b^
L*	27.20 ± 0.30^a^	27.80 ± 0.40^a^
a*	8.20 ± 0.20^a^	10.00 ± 0.10^b^
b*	7.40 ± 0.20^a^	8.30 ± 0.20^b^
ΔE	–	1.76 ± 0.14
C	11.00 ± 0.10^a^	12.80 ± 0.01^b^
*h*	42.30 ± 0.40^a^	38.70 ± 0.20^b^

Note: Control (no ultrasonic treatment); US (ultrasonic treatment). In the same column, different superscript letters represent significant differences (*p* < 0.05) according to the Tukey test.

### 3.4 Dissolution kinetics of proteins in LBP

The dissolution kinetics of LBPr was investigated with respect to experimental variables such as ultrasonic power, dual-frequency modes, sonication interval and treatment time, and a kinetic model for protein dissolution was developed to describe the dissolution process.

**Fig. 3:**
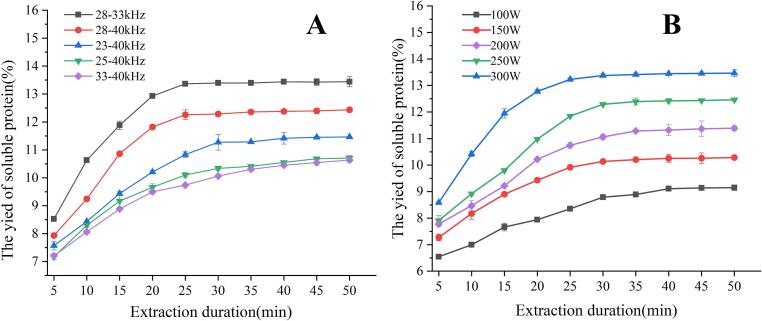
Relationship between sonication time and dual-frequency combination (A) and ultrasonic power (B).

As shown in Fig. 3 (A, B), the dissolution rate of LBPr varies greatly under different sonication conditions. The maximum dissolution rate of LBPr is 13.23 % after 25 min sonication (28–33 kHz and 300 W), suggesting that an appropriate ultrasonic conditions contribute to the protein dissolution in LBP. This is mainly due to that the turbulence and acoustic cavitation causing maximum cell wall disruption, leading to an increase in mass transfer rate and subsequently promoting protein release into the solvent [66]. Although the protein dissolution rate significantly increases within 20 min, it then rises slowly and reaches a flat horizontal, which is similar to the findings of Li et al*.* in extracting rice protein [38]. Therefore, it is not the longer the sonication time, the better.

#### 3.4.1 Analysis of the rate constant *k*

**Fig. 4:**
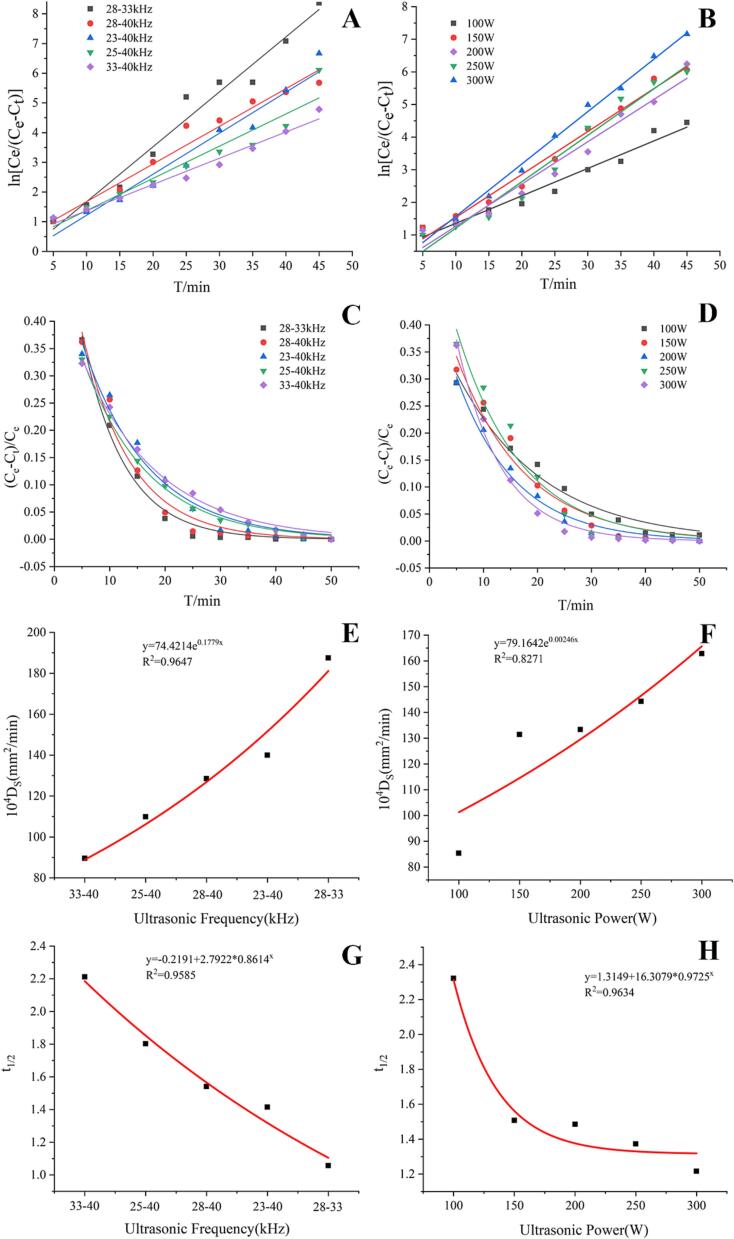
Kinetic correlation parameters of protein dissolution. Relationship between ln *[C_e_/(C_e_ − C_t_)]* and ultrasonic time under different dual-frequency combinations(A) and ultrasonic powers (B); Relationship between relative raffinate rate and ultrasonic time under different dual-frequency combinations (C) and ultrasonic powers (D); Relationship between *Ds* and dual-frequency combinations (E) and ultrasonic power (F); Relationship between *t_1/2_* and dual-frequency combinations (G) and ultrasonic power (H).

The rate constant *k* was calculated using Eq. (8), it represents the dissolution rate of LBPr, and the larger the *k* value, the faster the protein dissolves in LBP. As shown in Fig. 4 (A, B), the values of ln *[C_e_/(C_e_-C_t_)]* are fitted with the time *t*. It is found that there is an extremely strong linear relationship between ultrasound frequency modes and power, and all linear correlation coefficients (*R^2^*) of the fitted equations are higher than 0.92 (Table 4), indicating that the data fit well with the calculated values of the kinetic model and is suitable for predicting the dissolution process of LBPr. The *k*-value of the dual-frequency combination of 28–33 kHz is the highest. When the frequency is fixed, the *k*-value also gradually increases with the increase of ultrasound power; this could be explained by the fact that the increase of ultrasound power accelerating the process of solvent penetration, internal diffusion, external diffusion, making it easier for the internal matter to be released into the solvent, thereby promoting protein dissolution [67].

**Table 4:** Dissolution kinetics related parameters of *Lycium barbarum* protein.

		Rate constant				Relative raffinate rate		
		*k*	*b*	*R^2^*		*a*	*b*	*R^2^*
Power (W)	100	0.0842	0.5158	0.9591		0.4244	0.0626	0.9753
	150	0.1296	−0.0318	0.9613		0.4820	0.0921	0.9855
	200	0.1316	0.2228	0.9811		0.5083	0.0793	0.9628
	250	0.1423	−0.2149	0.9613		0.5965	0.084	0.9567
	300	0.1606	−0.0370	0.9938		0.6825	0.1213	0.9907
Frequency (kHz)	28–33 28–40 23–40 25–40 33–40	0.1849 0.1268 0.1381 0.1084 0.0883	−0.1766 0.4071 −0.1633 0.2905 0.4875	0.9728 0.9650 0.9563 0.9235 0.9784		0.7163	0.1305	0.9891
						0.6697	0.1134	0.9753
						0.5498	0.0841	0.9737
						0.5127	0.0853	0.9971
						0.4781	0.0730	0.9928

#### 3.4.2 Analysis of relative raffinate rate

Relative raffinate rate *y=(C_e_-C_t_)/C_e_* reflects the ratio of the undissolved component in the material to the mass concentration of the compound when the dissolution reaches equilibrium, and is fitted to time *t*. As shown in Fig. 4 (C, D), the relative raffinate rate rapidly decreases with time, however, when the time exceeds 30 min, the changes in the relative raffinate rate tend to stabilize and gradually reach the minimum, suggesting that prolonged ultrasonic time does not increase protein dissolution. The lowest relative raffinate rate was found at 28–33 kHz and 300 W, indicating that the dissolution of LBPr was maximized under these conditions. Similarly, the rate constant *a*, like *k*, increases with the increase of ultrasonic power (Table 4). The correlation coefficients *R^2^* were in the range of 0.95 to 0.99, and the accuracy of the curve fit is good, indicating that the LBPr dissolution process also follows the exponential model.

#### 3.4.3 Analysis of diffusion coefficient (*D_S_*)

The effective diffusion coefficient (*D_S_*) represents the protein diffusion rate within LBP particles. As shown in Fig. 4 (E, F), *D_S_* follows the exponential model at different frequencies and power, it increases significantly with increasing ultrasonic power. At constant power, selecting an appropriate ultrasonic frequency could significantly improve the diffusion rate of LBPr. It may be that different combinations of ultrasonic frequencies are more likely to form tiny cavitation bubbles in the pulp and collapse violently [68], thus increasing the diffusion of soluble LBPr in the pulp.

#### 3.4.4 Analysis of half-life period (*t_1/2_*)

The dissolution efficiency of LBPr can be reflected by the value of *t_1/2_*. As shown in Fig. 4 (G, H), the *t_1/2_* value decreases significantly with the increase of ultrasonic power. At 300 W and 28–33 kHz, the value of *t_1/2_* is the lowest, suggesting that higher power makes protein molecules move faster and effectively shortens dissolution time. In addition, different combinations of ultrasonic frequencies generate different types of physical fields, and the appropriate frequency modes are conducive to improving the dissolution efficiency. Therefore, it is reasonable to employ a nonstationary diffusion model to characterize, describe, and predict the dissolution process of LBPr. Ultrasonication (apparently 28–33 kHz, 300 W) decreased the mass transfer resistance between LBPr and solvent, the kinetic constant value increased. This may be related to the higher acoustic cavitation effect at 20–28 kHz (small bubble bursting), which generates higher pressure and temperature, as well as stronger excitation waves that impact the cell wall and promote the release of soluble protein into LBP, thus increasing the values of *y*, *k* and *D_S_* [39].

### 3.5 Effects of ultrasound on LBPr structure properties

#### 3.5.1 Uv–vis spectroscopy

**Fig. 5.1:**
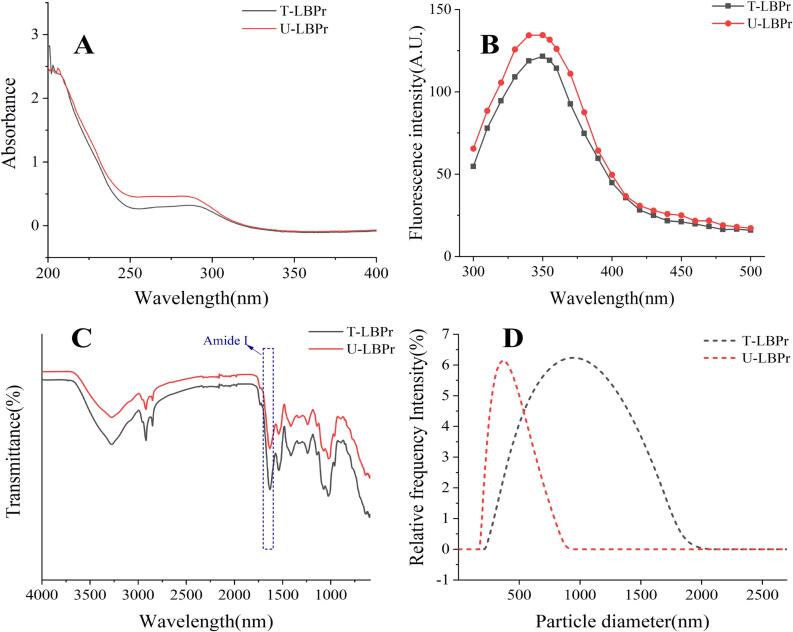
Structural Analysis of *Lycium barbarum* protein (LBPr), Ultrasonic treatment: U-LBPr; Traditional processing: T-LBPr. UV–Vis spectrogram (A), Fluorescence spectrogram (B), FT-IR spectrogram (C), Particle size (D).

As shown in Fig. 5.1A, compared with traditional method, treated with ultrasound enhances the absorption intensity, which is consistent with the results of Wen et al*.* [69]. This may be due to the fact that ultrasound may lead to the disruption of protein structure and expose more hydrophobic amino acid residues [70], inducing the refolding of protein molecules, which significantly changes the maximum absorption wavelength shifts of the amino acid residues, thus enhancing the UV absorption intensity [71].

#### 3.5.2 Fluorescence spectroscopy

Protein structure may be altered by the high temperature, pressure, and shear force generated by the collapse of cavitation bubbles during sonication [69]. Due to the presence of aromatic amino acid residues that are highly sensitive to environmental changes, fluorescence spectroscopy is often used to characterize the alterations in the tertiary structure of proteins [72]. As shown in Fig. 5.1B, the fluorescence intensity of U-LBPr is significantly higher than that of T-LBPr, which may be related to the hydrophobic interactions within the protein molecules, and the fluorescence spectra of U-LBPr show a blue shift, indicating that the tryptophan residues have shifted from a hydrophilic environment to a hydrophobic or less polar microenvironment [73].

#### 3.5.3 FT-IR spectroscopy

FT-IR spectrograms between 110 and 1700 cm^−1^ contained information about the conformation of protein peptides, the most valuable of which is the amide I band (spectra between 600 and 1700 cm^−1^). Amide I band is sensitive to the secondary structure changes, such as *α*-helix, *β*-turn, *β*-sheet, and random roll of proteins, and is therefore often used to identify secondary structure [74]. As shown in Fig. 5.1C, no significant differences in the amide I (1600–1700 cm^−1^) and amide II (1500–1600 cm^−1^) bands are found in the FTIR spectra. The secondary structure analysis of U-LBPr shows that, the content of irregular coil of U-LBPr significantly increases, while the contents of *α*-helix and *β*-helix significantly respectively decrease by 9.24 % and 24.70 % (Table 5), which are similar to current reports [75], [76]. *α*-helix is a kind of compact spatial structure in protein molecules, and the decrease in their proportion reflects that the destruction of hydrogen bonds by ultrasonic shear force, which stretches the structure of protein and exposes more active sites [77], [78].

**Table 5:** Structural and functional properties of *Lycium barbarum* protein (LBPr).

Group	*β*-turn	Randomcoil	*β*-sheet	*α*-helix	Zetapotential(mV)	Particlesize(nm)	
T-LBPr	33.23 %±0.21^a^	17.73 %±0.28^a^	23.98 %±0.32^a^	25.14 %±0.11^a^	−25.39 ± 1.38^a^	1057.23 ± 17.45^a^	
U-LBPr	30.16 %±0.14^b^	25.34 %±0.51^b^	25.15 %±0.47^b^	18.93 %±0.20^b^	−34.21 ± 0.99^b^	612.46 ± 10.14^b^	
	PDI	S_0_	Free SH	OHC(g/g)	WHC(g/g)	*Tp*(℃)	*ΔH*(J/g)
T-LBPr	28.46 ± 1.44^a^	418.57 ± 28.57^a^	8.41 ± 0.61^a^	2.41 ± 0.10^a^	3.15 ± 0.12^a^	63.06 ± 0.50^a^	1.79 ± 0.07^a^
U-LBPr	22.71 ± 1.92^b^	639.45 ± 7.83^b^	12.27 ± 0.56^b^	3.76 ± 0.08^b^	3.31 ± 0.15^a^	68.37 ± 0.15^b^	4.35 ± 0.12^b^

Note: Ultrasonic treatment: U-LBPr; Traditional processing: T-LBPr. Values were mean ± standard deviation (SD). In the same column, different superscript letters represent significant differences (*p* < 0.05) according to the Tukey test.

#### 3.5.4 Particle size and zeta potential

Particle size and distribution are the key indicators of protein aggregation [79]. As shown in Fig. 5.1D, the particle size of U-LBPr significantly reduces compared to that of T-LBPr. The average particle size of T-LBPr and U-LBPr is 1057.23 nm and 612.46 nm (Table 5), respectively, with a 42.10 % reduction in the average particle size and a significant reduction in the polydispersity index (PDI), which is similar to the report by Li et al. [80]. The absolute value of the zeta potential of U-LBPr is significantly higher, in agreement with the result of Lo et al*.* [81]. The higher the value of zeta potential, the better the stability of the system [82]. This may be due to the turbulence force and shear force generated by ultrasound, which cause larger protein aggregates to dissociate into smaller particles, narrow the particle size distribution, and expose more polar groups, resulting in the increase in the protein surface charge and less prone to polymerization, making the system more stable [83].

#### 3.5.5 SDS-PAGE analysis

**Fig. 5.2:**
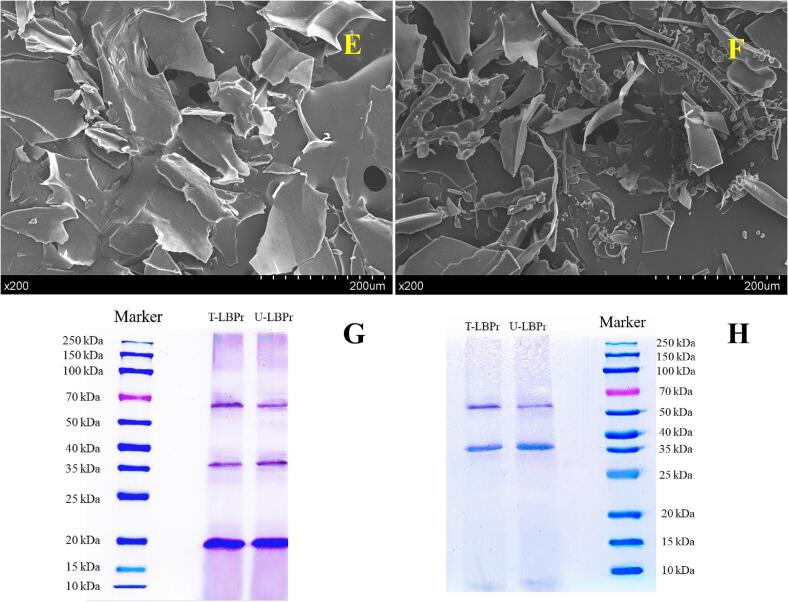
Structural Analysis of *Lycium barbarum* protein (LBPr), Ultrasonic treatment: U-LBPr; Traditional processing: T-LBPr. Microstructure of T-LBPr (E) and U-LBPr (F), Reducing (G) and non-reducing (H) SDS-PAGE of LBPr.

Reducing and non-reducing electrophoresis could reflect the molecular weight of LBPr. As shown in Fig. 5.2G and H, all samples exhibit three distinct bands with molecular weights ranging from 60 kDa to 70 kDa, 35–40 kDa, and 15–20 kDa, respectively, indicating that ultrasonic treatment did not affect the molecular weight distribution of LBPr, which was consistent with the results of scallop protein and sesame bran protein [48], [84]. This may be because the protein backbone could resist the physical forces generated by ultrasound, although the hydrogen bonds between the protein molecules are altered [85].

#### 3.5.6 Amino acid analysis

Amino acid composition is a key parameter determining the quality of the obtained protein [86]. Accordingly, the physicochemical and functional properties of proteins can be predicted from the information obtained from amino acid analysis [87]. As shown in Table 6, LBPr is rich in aromatic amino acids (Tyr, Phe), branched chain amino acids (Val, Ile, Leu) and Pro, which may explain its high antioxidant properties [88]. Furthermore, ultrasound-induced changes in amino acid ratios show that the content of total amino acids (TAA, 78.54 g/100 g) and essential amino acids (EAA, 41.94 g/100 g) of U-LBPr is significantly higher than that of T-LBPr. Furthermore, U-LBPr is rich in a higher proportion of hydrophobic amino acids (41.82 g/100 g), mainly including Leu (8.30 g/100 g), Gly (5.21 g/100 g), Ile (5.42 g/100 g), Met (1.22 g/100 g), and Pro (4.89 g/100 g). This may be due to the lossening of protein structure caused by acoustic-chemical effects, resulting in changes in secondary and tertiary structure of proteins and affecting the quantity and proportion of amino acids [89].

**Table 6:** Amino acid composition of *Lycium barbarum* protein (LBPr).

Animo acid	Content (g/100 g)	
	T-LBPr	U-LBPr
Asp	11.47	11.26
Thr	4.91	5.04
Ser	5.52	5.29
Glu	14.77	14.42
Gly	5.02	5.22
Ala	5.93	5.99
Cys	1.07	1.24
Val	5.69	5.69
Met	0.75	1.22
Ile	5.18	5.43
Leu	7.72	8.30
Tyr	4.37	4.35
Phe	5.00	5.07
His	3.28	3.42
Lys	7.93	7.76
Arg	5.86	5.59
Pro	4.52	4.89
Total	47.06	78.54
Hydrophobic amino acid	39.81	41.82
The hydrophilic amino acids	15.87	15.93
Total essential	40.47	41.94
Total non-essential	58.52	58.26
Branched chain amino acid	25.64	25.54
Aromatic amino acid	9.37	9.42

Note: Data shown are the mean ± SD, n = 3. Ultrasonic treatment: U-LBPr; Traditional processing: T-LBPr.

#### 3.5.7 Microstructure

The microstructure of LBPr was observed by SEM. As shown in Fig. 5.2(E, F), there are obvious differences between the microstructures of T-LBPr and U-LBPr. The texture of U-LBPr is dispersed into irregular fragments in a disordered manner, while the morphology of T-LBPr exhibits a larger lamellar structure with smoother surface, further confirming that sonication leads to a reduction in protein particle size.

#### 3.5.8 Surface hydrophobicity (S_0_) and free SH contents

As shown in Table 5, compared with T-LBPr (418.57), the surface hydrophobicity of U-LBPr (639.45) increased by 52.77 %. Furthermore, the free sulfhydryl content of U-LBPr (12.27 μmol/g) is significantly enhanced by sonication compared to that of T-LBPr (8.41 μmol/g). This could be attributed to the expansion and unfolding of the protein structure by ultrasound, from aggregated and dense to loose, as evidenced by the smaller particle size and altered morphology, which exposes the internal SH to the solvent [90].

### 3.6 Effects of ultrasound on LBPr functional properties

#### 3.6.1 Solubility

**Fig. 6:**
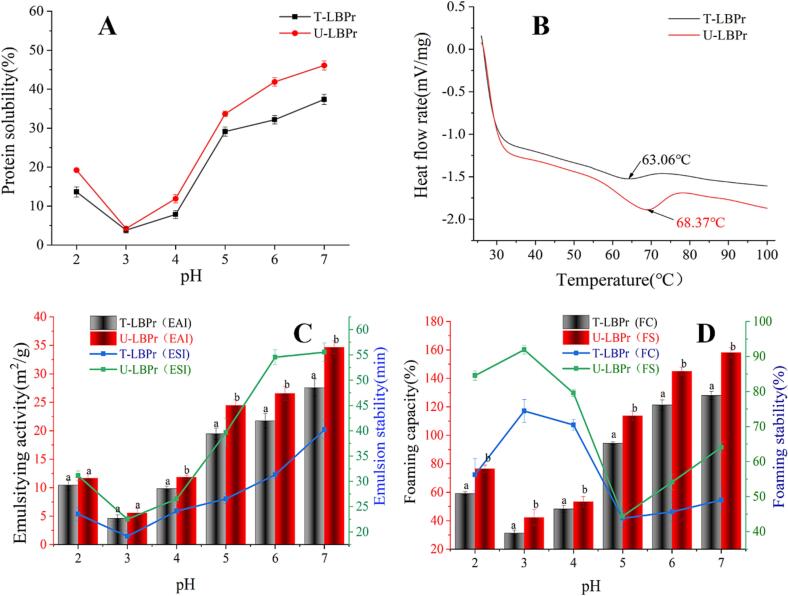
Functional properties of *Lycium barbarum* protein (LBPr), Ultrasonic treatment: U-LBPr; Traditional processing: T-LBPr. Solubility analysis (A), DSC curve (B), Emulsion activity and stability indices (C), Foaming capacity and stability (D).

The solubility of proteins largely depends on the pH of solution. At a pH close to the isoelectric point of a protein, the charge of ionizable groups decreases, leading to weakened charge repulsion behavior and internal molecules aggregation, which is unfavorable for protein solubilization [91]. As shown in Fig. 6A, there is no significant difference between the solubility of T-PBPr and U-PBPr at pH = 3. However, at other pH values, ultrasonication significantly improved the solubility of LBP (*p* < 0.05), especially at pH = 7, the solubility of U-LBPr increased by 23.32 % compared with T-LBPr. It may be related to the decrease in the particle size, the opening of *a*-helix, and the extended structure of protein [92].

#### 3.6.2 WAC and OAC

Oil absorption capacity is mainly affected by the protein type and hydrophobic residues. As shown in Table 5, the oil absorption capacity of U-LBPr (3.76 g/g) is higher than that of T-LBPr (increased by 56.02 %), which may be related to the increase in the proportion of hydrophobic amino acids and S_0_ caused by ultrasonic treatment. However, no significant improvement in protein water absorption capacity was found in this study, it is consistent with the report of Li et al*.* on brewer's dregs protein [93].

#### 3.6.3 Emulsion activity and stability indices

Protein emulsification is the ability of protein to form stable emulsion between immiscible oil and water phases, which plays an important role in food processing, and can be described by EAI and ESI. As shown in Fig. 6C, the emulsifying activity of LBPr increases with the increase in pH. Compared with T-LBPr, the EAI of U-LBPr at pH = 7 is 44.65, increased by 22.03 % (*p* < 0.05); and the ESI of U-LBPr at pH = 6 is 54.56, increased by 74.12 %. These results suggest that ultrasonication can significantly improve the capacity of protein to form and stabilize emulsion. Studies have shown that the protein adsorption and emulsification activity at the oil–water interface can be improved by exposing the hydrophobic groups of proteins [94]. Therefore, it can be concluded that ultrasonic shearing destroys the molecular structure of the protein, reduces the particle size and increasing the surface charge, resulting in protein emulsions with smaller particle sizes and improved emulsion stability [95].

#### 3.6.4 Foaming capacity and stability

Foaming capacity (FC) is positively correlated with protein solubility, as shown in Fig. 6D, the FC of U-LBPr is significantly higher than that of T-LBPr (*p* < 0.05), especially at pH = 7 the foaming stability increased from 49.04 % to 64.07 % (increased by 30.65 %). This may be closely related to that ultrasonication unfolds proteins molecules, exposing more hydrophobic groups and increasing air-protein interactions [23].

#### 3.6.5 Thermal properties analysis

DSC provides specific information on thermal properties, such as denaturation temperature and denaturation enthalpy, and can be used to study the protein changes with temperature [96]. *Tp* reflects the temperature required for protein denaturation and *ΔH* shows the amount of heat required to induce protein denaturation. As shown in Table 5, both *Tp* and *ΔH* of U-LBPr are significantly increased, suggesting sonication improves the thermal stability of LBPr, which may be caused by the change in the number of protein hydrogen bonds and the decrease in particle size [85]. Besides, sonication improves the surface hydrophobicity of the protein, which leads to a decrease in surface tension and stiffness, thus increasing the thermal stability [87].

Due to the uniform sound field created by slit dual-frequency ultrasound, the cavitation threshold is reduced, and most of the energy when the cavitation bubbles ruptured will be converted into the kinetic energy of the jet beam [106], which directly acted on the surface of the LBPr, leading to the loosening and homogenization of the protein fragments, and the reduction of particle size. Moreover, ultrasound led to the exposure of hydrophobic groups, weakened hydrogen bonding and the change of secondary structure from ordered to disordered, and these conformational changes were conducive to the enhancement of the emulsification ability, foaming performance, and thermal stability of the protein [65].

### 3.7 Effects of ultrasound on LBPr antioxidant properties

**Fig. 7:**
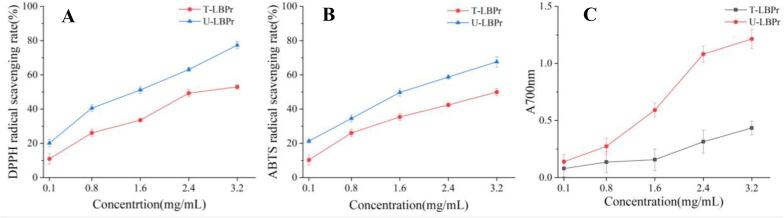
Antioxidant activity of *Lycium barbarum* protein (LBPr), Ultrasonic treatment: U-LBPr; Traditional processing: T-LBPr. DPPH• radical scavenging rate (A), ABTS+• radical scavenging rate (B), Reducing power (C).

The antioxidant properties of U-LBPr obtained under optimal conditions were compared with those of T-LBPr by traditional method. As shown in Fig. 7, the antioxidant capacity of U-LBPr and T-LBPr gradually increases with increasing concentration, and the scavenging rate is positively correlated with the concentration. When the concentration is 3.2 mg/mL, the DPPH• and ABTS^+^• radical scavenging rate, and the reducing power of U-LBPr respectively increase by 46.01 %, 35.52 %, and 143.08 % compared with that of T-LBPr. These results indicated that ultrasonic treatment significantly enhanced the antioxidant properties of LBPr. Similar results were obtained by Wen, Wang, Lian, and Zhao [96], [97], [98]. This may be due to the high-intensity shear and cavitation effect of sonication, which weakens the van der Waals forces, hydrogen bonds, and other non-covalent bonds of LBPr, and increases the content of irregular curls, leading to the stretching of protein structure and providing more free radical reaction sites [69], which is consistent with the results of altered secondary structure by FTIR. In addition, protein particle size significantly affects antioxidant properties [99], and sonication significantly reduces LBPr particle size, exposing more hydrophobic groups. Furthermore, the increase in sulfur-containing amino acids such as methionine and cysteine as well as aromatic amino acids after ultrasonication enhances the free radical scavenging capacity by providing protons, which may be one of the reasons for the enhanced antioxidant capacity of U-LBPr [100]. All these indicate that the structure of U-LBPr was modified and showed better antioxidant capacity.

### 3.8 Establishment of in situ real-time monitoring model of LBPr dissolution process by NIR spectroscopy

**Fig. 8.1:**
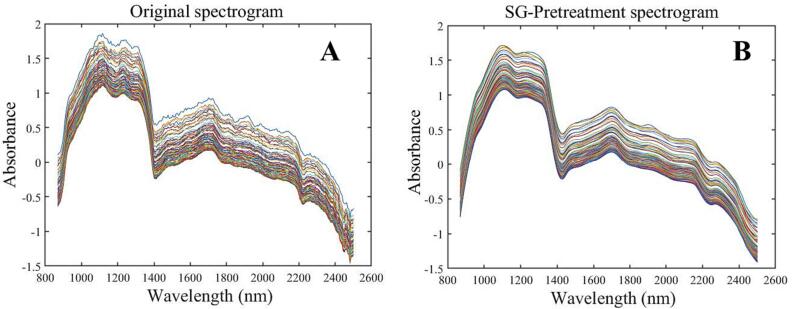
Original spectrogram (A) and SG-Pretreatment spectrogram (B).

A real-time protein dissolution monitoring model for ultrasonic pulping was developed by combining NIR spectroscopic information with LBPr content measured by offline-sampling during ultrasonic process. Fig. 8.1A shows the original spectrogram during the acquisition process. The raw NIR spectra acquired by in-situ monitoring system contain information unrelated to protein structure changes, such as background noise, baseline drift, and solute scattering [26], In order to obtain stable, reliable and accurate models, some pre-processing of the original spectra is necessary [101]. In this paper, the original spectra were preprocessed using standard normal variate transform (SNV), Savitzky-Golay smoothing (SG), First derivative (1st) and Second derivative (2nd), and PLS was used to establish the corresponding quantitative analysis model to determine the optimal preprocessing method. The root mean square error of cross-validation (*RMSECV*), root mean square error of prediction (*RMSEP*), calibration set correlation coefficient (*Rc*), and prediction set correlation coefficient (*Rp*) were used to evaluate the accuracy and stability of each model. If *Rp* and *Rc* approximate 1 and *RMSECV* and *RMSEP* approximate zero, it means that a better and more stable model has been constructed [102]. The results are shown in Table 7. After pre-processed by SG method, the filter function smooths the spectral curves and retains the effective information at low frequency, while the noise at high frequency is removed, thus improving the signal-to-noise ratio [103]. Model *Rc* and *Rp* were 0.9692 and 0.9755 respectively, which were the highest among five preprocessing procedures, while *RMSECV* (2.962) and *RMSEP* (1.494) were the lowest. These results indicated that the SG-preprocessed raw NIR spectra can better predict the dissolution process of LBPr. Therefore, the SG-preprocessed spectra were selected for the subsequent modeling analysis (Fig. 8.1).

**Table 7:** Comparison of different pretreatment methods and analysis results of Si-PLS model with LBPr dissolution rate under different sub-interval numbers.

Parameters	Pretreatment method	Number of subintervals	Selected subintervals	PC_S_	*Rc*	*RMSECV*	*Rp*	*RMSEP*
The dissolution rate of LBPr (%)	SNV	–	–	3	0.9585	3.318	0.9406	1.431
	**SG**	–	–	**4**	**0.9692**	**2.962**	**0.9755**	**1.494**
	1st Der	–	–	9	0.9588	3.161	0.9688	1.610
	2nd Der	–	–	5	0.8471	5.767	0.8916	2.453
	SNV-1st	–	–	9	0.9612	3.066	0.9719	1.534
	SG-1st	–	–	8	0.9244	4.301	0.9663	1.398
	SNV-2nd	–	–	7	0.9309	4.005	0.9648	3.045
	SG-2nd			4	0.9668	3.045	0.9715	1.613
		10	[3], [4], [6], [7]	8	0.9722	2.828	0.9681	1.591
		11	[1], [3], [7], [8]	10	0.9796	2.412	0.9689	1.614
		12	[3], [8], [9], [10]	10	0.9776	2.539	0.9796	1.368
		13	[1], [3], [8], [10]	10	0.9785	2.456	0.9804	1.281
	Analysis of the Si-LPS model after the SG-preprocessed	14	[2], [6], [8], [11]	10	0.9773	2.515	0.9752	1.473
		15	[4], [5], [12], [15]	10	0.9822	2.251	0.9626	1.873
		16	[1], [2], [5], [10]	9	0.9760	2.607	0.9863	1.301
		17	[2], [3], [4], [13]	10	0.9793	2.402	0.9677	1.602
		18	[6], [9], [11], [14]	10	0.9794	2.424	0.9457	2.386
		19	[5], [12], [13], [14]	10	0.9770	0.568	0.9725	1.571
		20	[4], [6], [7], [14]	8	0.9778	2.502	0.9692	1.508
		21	[2], [4], [5], [16]	10	0.9789	2.445	0.9666	1.608
		**22**	[2], [6], [14], [17]	**9**	**0.9835**	**2.174**	**0.9841**	**1.206**
		23	[3], [6], [17], [18]	10	0.9815	2.286	0.9733	1.507
		24	[3], [8], [14], [18]	10	0.9801	2.363	0.9567	1.943
		25	[3], [8], [14], [19]	10	0.9796	2.409	0.9654	1.822
		26	[5], [7], [9], [18]	9	0.9805	2.348	0.9675	1.548
		27	[6], [11], [20], [21]	10	0.9806	2.343	0.9786	1.232
		28	[4], [7], [21], [22]	10	0.9849	2.066	0.9691	1.601
		29	[4], [14], [21], [22]	10	0.9809	2.317	0.9360	2.747
		30	[6], [7], [22], [23]	9	0.9786	2.465	0.9681	1.398

Note: PCs refer to principal components; *Rc* and *Rp* refer to the correlation coefficient of the calibration set and prediction set, respectively; *RMSECV* and *RMSEP* refer to the root mean square error of cross-validation and prediction, respectively.

**Fig. 8.2:**
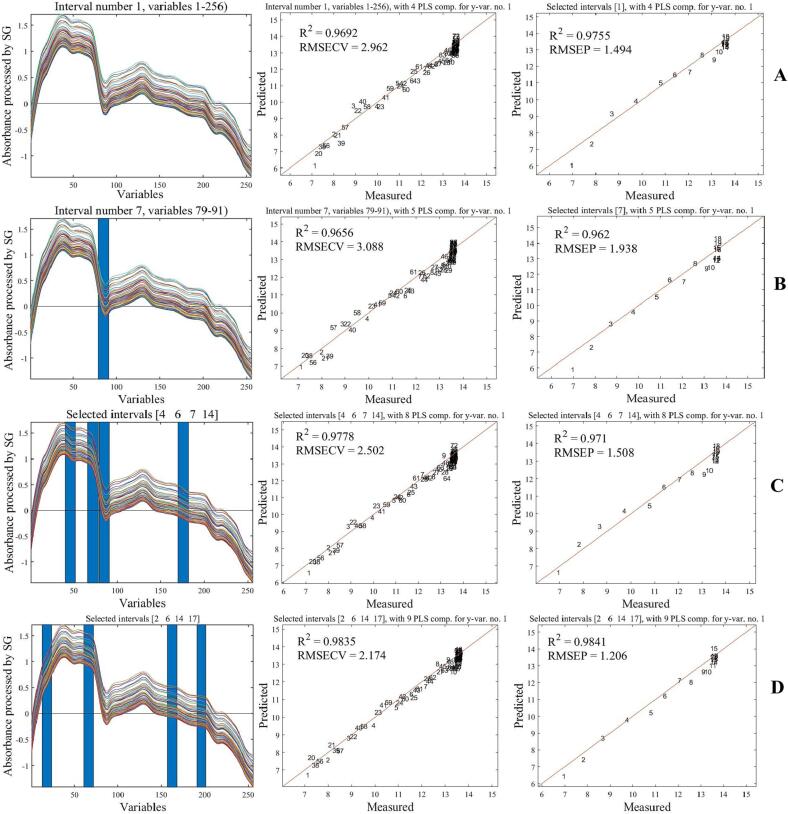
In-situ monitoring of protein dissolution rate in *Lycium barbarum* pulp(LBP) sample. Calibration set and prediction set of PLS model (A), Calibration set and prediction set of i-PLS model (B), Calibration set and prediction set of Si-PLS model (C), Calibration set and prediction set of the optimal joint subinterval Si-PLS model (D).

After spectral preprocessing, in addition to the useful spectral information, there are still some noise and unimportant information. Therefore, it is necessary to select the best features from the measured spectra. To characterize the spectral data, partial least squares (PLS), interval partial least squares (i-PLS), and synergy interval partial least squares (Si-PLS) were used to screen the spectral intervals. The filtered feature information was linked and modeled with the chemical values of protein content detected in real-time synchronization [104]. The quantitative models were constructed by PLS, i-PLS and Si-PLS (Fig. 8.2), and the magnitude of their *Rc* and *Rp*, *RMSECV* and *RMSEP* values were compared. The model constructed by Si-PLS modeling method had a corrective model *Rc* = 0.9778, *RMSECV* = 2.502, a predictive model *Rp* = 0.9710 and *RMSEP* = 1.508. Compared with the other two modeling methods, Si-PLS produced models with the highest accuracy and predictability. This may be that when modeling spectral data with the Si-PLS modeling method, the whole spectrum was divided into several subintervals, and then several subintervals were combined for feature information screening, which could eliminate the useless information of redundant spectra as well as screen the favorable feature information [105].

The entire spectrum was then divided into 10–30 intervals and four sub-intervals were combined for modeling. The results of Si-PLS model for different sub-intervals were shown in Table 7. The spectra were divided into 22 intervals with a principal component of 9. The *Rc* (0.9835) and *Rp* (0.9841) were the highest, and the *RMSECV* (2.174) and *RMSEP* (1.206) were the lowest, indicating that the Si-PLS model under the optimal combination of intervals [2], [6], [14], [17] was more highly correlated and better at predicting the content of soluble LBPr. The corresponding spectral regions were 972.73–1045.45 nm, 1263.64–1336.36 nm, 1772.72–1845.45 nm, and 2063.63–2136.36 nm. Among them,1263.64–1336.36 nm and 1772.72–1845.45 nm were the mid-wave near-infrared spectral region, reflected the double frequency information of hydroxyl (–OH) and amino (–NH) groups. The spectral band 2063.63–2136.36 nm was the long-wave near-infrared spectral region, reflected the combined frequency information of hydroxyl (–OH) and amino (–NH) groups. The quantitative analysis models established for LBPr dissolution under the optimal joint subinterval, respectively, and the scatter plots between the measured and predicted values of the samples in the calibration and prediction sets of the model are shown in Fig. 8.2D, with determination coefficient *Rc* = 0.9835 and *RMSECV* = 2.174 for calibration model, and determination coefficient value *Rp* = 0.9841 and *RMSEP* = 1.206 for prediction model, the model can realize real-time online monitoring of dissolution in ultrasound-assisted pulping process.

## 4 Conclusion

In the face of the difficulties of the traditional pulping industry, ultrasound-assisted pulping is an effective way to improve the processing efficiency and economic benefits of the fresh fruit of *Lycium barbarum*. Under the optimal conditions of dual frequency ultrasound, the content of LBPr and other active ingredients could be significantly improved. Ultrasonication significantly changed the amino acid ratio and secondary structure of LBPr, decreased the particle size of the protein, and increased the surface hydrophobicity of the protein, which was closely related to the improvement of its antioxidant activity, and caused the improvement of LBP efficacy at the same time. In addition, the functional properties of LBPr, such as thermal stability, foaming, and emulsification, were significantly improved by ultrasound, which provides a possibility for the development of LBPr. Furthermore, the dissolution law was further elucidated by establishing a kinetic model of dissolution during ultrasound-assisted pulping. On this basis, in order to improve the intelligent control of the pulping process and realize the rapid judgment of the end point, a real-time monitoring model for the ultrasound-assisted pulping process of fresh berries was established. These results indicated that slit dual-frequency ultrasound has great potential for improving the quality of LBP, which may provide a theoretical basis for the establishment of an intelligent control system in the industrialized production of LBP and the functional development of LBPr. However, the molecular mechanism of the antioxidant activity of LBPr and the detailed correlation between LBPr structure and activity need to be further investigated.

## CRediT authorship contribution statement

**Shuhan Liu:** Investigation, Writing – original draft. **Tianyu Kong:** Investigation, Validation. **Yuqin Feng:** Methodology, Validation. **Yanli Fan:** Investigation, Validation. **Junwei Yu:** Investigation, Validation. **Yuqing Duan:** Supervision, Project administration. **Meihong Cai:** Investigation, Validation. **Kai Hu:** Investigation, Validation. **Haile Ma:** Investigation, Validation. **Haihui Zhang:** Funding acquisition, Writing – review & editing.

## Declaration of Competing Interest

The authors declare that they have no known competing financial interests or personal relationships that could have appeared to influence the work reported in this paper.
